# Can Meat and Meat-Products Induce Oxidative Stress?

**DOI:** 10.3390/antiox9070638

**Published:** 2020-07-20

**Authors:** Adrián Macho-González, Alba Garcimartín, María Elvira López-Oliva, Sara Bastida, Juana Benedí, Gaspar Ros, Gema Nieto, Francisco José Sánchez-Muniz

**Affiliations:** 1 Nutrition and Food Science Department (Nutrition), Pharmacy School, Complutense University of Madrid, 28040 Madrid, Spain; amacho@ucm.es (A.M.-G.); sbastida@ucm.es (S.B.); 2 Pharmacology, Pharmacognosy and Botany Department, Pharmacy School, Complutense University of Madrid, 28040 Madrid, Spain; a.garcimartin@ucm.es (A.G.); jbenedi@ucm.es (J.B.); 3 Departmental Section of Physiology, Pharmacy School, Complutense University of Madrid, 28040 Madrid, Spain; elopez@ucm.es; 4 Department of Food Technology, Food Science and Nutrition, Faculty of Veterinary Sciences, Regional Campus of International Excellence “Campus Mare Nostrum”, Espinardo, 30071 Murcia, Spain; gros@um.es

**Keywords:** meat, metabolism, oxidation, oxidative stress

## Abstract

High meat and meat-products consumption has been related to degenerative diseases. In addition to their saturated fatty acids and cholesterol contents, oxidation products generated during their production, storage, digestion, and metabolization have been largely implicated. This review begins by summarizing the concept of meat and meat-products by the main international regulatory agencies while highlighting the nutritional importance of their consumption. The review also dials in the controversy of white/red meat classification and insists in the need of more accurate classification based on adequate scores. Since one of the negative arguments that meat receives comes from the association of its consumption with the increase in oxidative stress, main oxidation compounds (malondialdehyde, thermaloxidized compounds, 4-hydroxy-nonenal, oxysterols, or protein carbonyls) generated during its production, storage, and metabolization, are included as a central aspect of the work. The review includes future remarks addressed to study the effects meat consumption in the frame of diet–gene interactions, stressing the importance of knowing the genetic variables that make individuals more susceptible to a possible oxidative stress imbalance or antioxidant protection. The importance of consumed meat/meat-products in the frame of a personalized nutrition reach in plant-food is finally highlighted considering the importance of iron and plant biophenols on the microbiota abundance and plurality, which in turn affect several aspects of our physiology and metabolism.

## 1. Introduction

Currently, food, in addition to being a source of energy, nutrients and bioactive compounds assuring growth and development, is perceived as a key factor that influences body functions and may help to prevent some degenerative diseases [1]. Although diet composition and quality significantly differ between countries and/or World regions, meat has generally been considered a central food item that once consumed, assures several nutritional compounds needed for the correct homeostasis at any age. Nonetheless, despite being an excellent source of nutrients and bioactive compounds, there are epidemiological concerns linking excessive meat consumption with increased risk of various chronic diseases, such as cardiovascular diseases (CVD) and some types of cancer, particularly colorectal cancer [2,3]. However, recent research data highlight the need to performed accurate meta-analysis collecting and pooling dietary data from different cohorts analyzing each diet-disease pair after adjusting for same confounders [4].

In addition, the relationship between meat consumption and degenerative diseases has been wedged by making inadequate generalizations, forgetting that “meat” is an ample concept that refers to the edible part of any mammal, as we have to take into account that mammal is also an ample concept covering terrestrial and marine mammals. The last one includes animals such as seals and whales, which normally are not consumed Western countries. In fact, it has to keep in mind that cross-sectional studies, performed at a certain time, cannot be directly used to relate cause and effect. More than 50 years ago, Hill [5] listed nine types of major evidences that could help reaffirm the results of observational studies: consistency, specificity, temporality, biological gradient, plausibility, coherence, experimentation and analogy. The Hill’s postulates are accepted and considered by both those who are in favor as for those who are in against using observational studies to draw conclusions about causation. Thus, the possible negative effects of meat are highly dependent on the nutritional status of consumers and the plurality of the dietary compounds eaten together with meat [2,6]. For instance, many experiments designed to mimic the nutrient loads in current westernized diets did not include potential biologically active protective compounds present in whole foods, limiting the evidence to confirm a mechanistic link between the intake of red meat as part of a healthy dietary pattern and colorectal cancer risk [7]. Although different mechanisms have currently been suggested to explain this association, the impact of meat consumption, especially red meat, is still a debate within the scientific community [7]. However, the common factor that could help to understand this relationship is the role of oxidative stress in the etiology and/or progression of these diseases [8].

Meat is a food rich in protein, lipids, and heme iron, whose composition makes it highly perishable as a consequence of the chemical and enzymatic processes that take place during production and storage. Lipids and proteins are susceptible to oxidation, being responsible for the loss of organoleptic and nutritional properties in this food type [9]. Additionally, the presence of transition metals such as heme iron greatly contributes to the formation of these undesired products. Among the oxidized products of lipid origin are malondialdehyde (MDA), 4-hydroxy-nonenal (4-HNE), 4-hydroxy-hexenal (4-HHE) or oxysterols, while those originating from proteins are mainly protein carbonyls. However, fat triglycerides in meat in presence of metal transition and oxygen suffer oxidation, oligomerization and hydrolysis opening discussion in this important topic [10]. The gastrointestinal tract is the gateway for oxidized food compounds produced during processing and storage of meat, but they can also originate during digestion and metabolization. Specifically, the stomach appears to be an excellent environment for meat lipid peroxidation, having observed a greater formation of lipid hydroperoxides, MDA and 4-HHE both in the stomach content and in plasma or urine after meat consumption [11,12,13]. Some studies have shown that the amount of oxidation compounds reaching the colon is approximately 50% less than that available at the small intestine, which is clearly indicative of a significant absorption rate [8]. The available literature indicates that a part of the diet oxidation compounds is absorbed in the lymph or directly into the bloodstream, being able to induce oxidative damage in biological systems [14,15].

Furthermore, in the frame of the precision diet/nutrition concept, a strong effort is being made to find out how the most convenient food items should be combined to assure good functionality and to decrease the degenerative disease risk of any given person considering the genoma-diet interaction [16,17,18]. Given this great interest, very active research is being performed to find out about, develop, and test numerous functional foods aimed at increasing the nutritional value and exerting some beneficial effects on certain diseases [19,20,21]. For this, it is first necessary to know the oxidative processes that can take place in meat and meat-products from their processing to its consumption. The present manuscript has the objective to describe the main oxidative processes that take place in meat and meat-products during their production, storage, cooking, digestion, and metabolization, which may be involved in the origin and development of more prevalent chronic degenerative diseases.

## 2. Meat and Meat-Products

Before starting to deal with the subject of this in-depth review, it is necessary to know the definition of meat concept by the main regulatory agencies at the European and American levels. According to the Codex Alimentarius Glossary [22], meat is the edible part of any mammal. This general definition gives a clear idea of the complexity of the topic as various parts of different various mammals differing in age, familiae, gender, species, varieties, feeding, etc., can be included in this general definition. For this reason, we would like to introduce a more detailed definition by the European Commission and the American Code of Federal Regulations.

Due to the extensive terminology on meat and meat-products, the American Meat Science Association (AMSA) developed in 2018 a standardized lexicon of terms that allows the precise description and classification of meat and meat-products to minimize confusion when transmitting meat-related information. Thus, AMSA defines meat as “skeletal muscle and its associated tissues derived from mammalian, avian, reptilian, amphibian, and aquatic species harvested for human consumption. Edible offal consisting of organs and non-skeletal muscle tissues also are considered meat” [23]. However, several authorities have their own definitions about meat and meat products, most times addresses to define meat and/or meat products normally consumed in each country’s sphere of influence.

Thus, the European Commission, according to Regulation (EC) No 853/2004 [24], understands by meat “edible parts of the animals referred in these three groups (*sic*): (a) Fresh meat of domestic and wild ungulates; (b) Game meat, and (c) Meat-products”.

(a) Fresh meat of domestic and wild ungulates: refers to meat that has not undergone any preserving process other than chilling, freezing or quick-freezing, including meat that is vacuum-wrapped or wrapped in a controlled atmosphere. The definitions of “meat” and “fresh meat” are laid down in Annex I of Regulation (EC) No 853/2004. Fresh meat of domestic and wild ungulates includes fresh meat of for example: bovine animals (including buffalo and bison); ovine and caprine animals; porcine animals; solipeds.

(b) Game meat: game animals refer to land mammals and birds—either in the wild or farmed—that are not normally considered to be domestic animals (the following animals are specifically excluded: bovines, domestic swine, sheep and goats, domestic solipeds, domestic fowl, turkeys, guinea-fowl, ducks, and geese). Fresh game meat must fulfil the animal health requirements laid down in the legislation applicable to each classification of game animal. This classification is based on both the species of animal and its origin. Therefore, there is a clear distinction made between: fresh meat from wild game, and fresh meat from farmed game.

(c) Meat-products: are defined as processed products, resulting from the processing of meat or from the further processing of such processed products, so that the cut surface shows that the product no longer has the characteristics of fresh meat.

Instead, the American Code of Federal Regulations does not make these distinctions and defines meat as “the part of the muscle of any cattle, sheep, swine, or goats which is skeletal or which is found in the tongue, diaphragm, heart, or esophagus, with or without the accompanying and overlying fat, and the portions of bone (in bone-in product such as T-bone or porterhouse steak), skin, sinew, nerve, and blood vessels which normally accompany the muscle tissue and that are not separated from it in the process of dressing. As applied to products of equines, this term has a comparable meaning” [25]. This document also defines meat-products, as “any article capable of use as human food which is made wholly or in part from any meat or other portion of the carcass of any cattle, sheep, swine, or goats, except those exempted from the definition as a meat food product by the Administrator in specific cases or by the regulations in part 317 of this subchapter”. Thus, the European Commission and the American Code of Federal Regulations do not include in their meat definitions any reference to marine mammals.

## 3. Production, Consumption, and Nutritional Importance of Meat and Meat-Products

Meat, meat-products, and other goodness of animal origin have greatly increased in number in the last century, especially during the last six decades all over the World [26]. If we look at the 2017 figures, overall world meat production increased by 1.25% to 323 million tons, with a predominant increase in bovine and poultry meats compared to pork and sheep meat. In fact, annual meat production is projected to increase by about 150 million tons in three decades (from 218 million tons in 1997–1999 to 367 million tons by 2027–2030) [26]. This increase is primarily due to two main factors, the increasing pressure on the livestock sector to meet the growing demand for high-value animal protein and improving productivity in developing countries. According to the Food and Agriculture Organization of the United Nations (FAO) and its database (FAOSTAT) the world’s livestock sector has been growing at an unprecedented rate due to a combination of population growth, rising incomes, and urbanization. However, this upward trend is expected to start to slow down, mainly as a result of lower production and consumption in Europe [26]. All these predictions could be clearly modified by health crises such as the current Covid-19.

Taking into account the consumption data of meat and meat-products represented in Table 1, we realize the importance of not generalizing in the concept of meat. Each country presents an eating pattern that is conditioned by reasons of culture and availability, which will directly have an impact on health. Nonetheless, it has to be pointed out that the declared consumption is just apparent and in some way misleading, as the “actual” meat consumption (the meat that we really eat) as that of other dietary compounds (e.g., vegetables) represents 33–50% of the apparent one that consider some not suitable part for consumption or does not take into account just wasting, that tend to be higher in the high income [27].

The main components of meat are water (60–80%), protein (16–25%) (approximately 40% of its amino acids are essential), and fat (1–30%). There are also small amounts of non-protein nitrogenous substances (free amino acids, peptides, creatine, nucleotides, etc.), carbohydrates, lactic acid, vitamins (thiamine, niacin, retinol, and vitamins B_6_ and B_12_), small amount of vitamin D and minerals (e.g., heme iron and Zn of high bioavailability) and others which are no less important such as P, Se, Na, K y Co [29]. Red meat is also a source of lipoic acid. However, these proportions vary depending on the animal, age, sex, diet, and anatomical areas analyzed [30].

The World Health Organization (WHO) already mentioned that meat and meat-products (sic) “not only provide high-value protein but are also important sources of a wide range of essential micronutrients, in particular minerals such as iron and zinc, and vitamins such as vitamin A”. For the large majority of people in the world, particularly in developing countries, livestock products remain a desired food for nutritional value and taste, being one of the causes of the increase in its production and consumption [31]. It should be noted that meat and meat-products are the main source of vitamin B_6_ and the second source of niacin [3]. Thus, the absence of meat in the diet of some countries makes it difficult to achieve the recommended intakes of vitamin B group. However, these figures could not be extrapolated to other world regions since diet and the type of meat consumed varies markedly between different countries (Table 2). Anyhow, its consumption can contribute partially to achieving the recommended dietary intakes of vitamins and minerals [3,32,33,34,35]. 

At present, there exists great concern about the intake of red and/or processed meat, as many studies have associate the intake of these products with the incidence and prevalence of chronic diseases such as obesity, type 2 diabetes mellitus (T2DM), CVD, and different types of cancer [2,3]. As a result of these studies, many health-related agencies have recommended restricting the intake of these products [36]. Nonetheless, we believe that these recommendations, made under the protection of health objectives, are not objective enough and deserve some degree of criticism and discussion, as different publications include [2,37,38,39]. In fact, some publications demand to perform new meta-analysis collecting and pooling dietary data from different cohorts analyzing each diet-disease pair after adjusting for same confounders [38]; other based in five de novo systematic reviews that considered certainty in the evidence, the magnitude of potential benefits and harms, and explicit consideration of people’s values and preferences have recently been made a set of recommendations on red meat and processed meat consumption. The panel recommendations were developed by using the Nutritional Recommendations (NutriRECS) guideline development process and suggest that adults continue current unprocessed red meat consumption (weak recommendation, low-certainty evidence). Similarly, the panel suggests adults continue current processed meat consumption (weak recommendation, low-certainty evidence) [4]. In addition, many epidemiological approaches based on meta-analyses of prospective observational studies adjust for total energy intakes their statistical models do not permitting to define risk in terms of absolute levels of exposure. Thus, the relative risk of change in each component of diet depends on the other components for which it is substituted, fact that is not generally specify in publications. Additionally, the effect size of the individual dietary factors might be overestimated, as the intake of healthy dietary factors are generally positively correlated with each other and inversely correlated with harmful dietary factors. These facts highlight the need to analyze each diet–disease pair, quantifying the effect size after adjusting for same confounders. In addition, most cohorts have been made without considering that suboptimal diet is an important preventable risk factor for non-communicable diseases [38].

Oxidation has been defined as an important factor for several chronic degenerative diseases [41]. Due to relatively high iron content of meat and the central role of iron as prooxidant [42], several works have associated red meat consumption with CVD development and some types of cancers, [43,44,45]. Nonetheless, this relationship has recently been criticized due to unacceptable generalization and because several points still pending verification. Thus, the problem of this association partially derives from the misuse of the terms “red” or “white” meats, being a misleading source due to their generic use both in the general population and in the scientific community. Currently, we still do not know how to differentiate which product could be considered red meat and which could not, and even within the same animal species we can find meat fractions classified as red and others as white. It may seem that the simplest classification in color terms seems also related to the iron content (Table 3). Nonetheless, it has to be taken into account that the amount of heme iron seems associated to the age of animals, as older animals have less moisture, leading any generalization to wrong conclusions. For example, in Spain, although Suckling, Recental, and Paschal lambs are available, due to organoleptic properties such as taste and flavor, the Suckling lamb is by far the most consumed and preferred by the Spanish people, clearly determining differences observed on heme iron between Spain and American ground lamb (Table 3). Furthermore, Spanish lamb heme iron content is rather close to that of Chicken breast clearly accepted as white meat. Moreover, when talking about poultry meat such as turkey, clear location has to be made as myoglobin content is higher in the legs than in the wings, as they hardly fly and their muscular activity is located in the legs, whose greater supply of oxygen is responsible for the redder meat. Thus, the extrapolation of results of heme iron should be carefully done, after considering the age and the meat part of the animals consumed or studied.

In addition, it has to be pointed out that there are no unique and unified criteria distinguishing red and white meats, since they have been classified according to their myoglobin concentration (heme iron) but also to lipid profile, mitochondrial densities, physiology of muscle fiber and/or in response to physiological changes during metabolism and/or postmortem proteolysis. Therefore, from here we are critical and request to accurately describe the parameter of real interest when communicating scientific information and not using the broad concept of red or white meat [23,46].

However, in relation to oxidation, what is the most determining factor? Current evidence indicates that iron is an important factor in the formation of oxidized compounds through the Fenton reaction, as summarized by Van Hecke et al. [8]. However, if we put on a scale the heme iron implication or the food lipid composition, it would be the availability of oxidizable substrates, such as lipids, that would clearly determine the meat oxidation levels [47,48,49]. Thus, we insist on the urgent need to perform ample controlled studies considering meat major factors (e.g., level of PUFA, heme iron and different meat protein with antioxidant/prooxidant properties) to find out a sort of ponderate oxidation score that allows us to classified meat accurately.

## 4. Meat Consumption as a Source of Oxidative Stress

Oxidative stress has been defined as a critical factor engaged in the origin of most degenerative diseases [41]. Diet has been found to contribute to the pro-oxidant and antioxidant balance as both the deficiency or the high consumption of some foods and nutrients are highly implicated [50].

Although meat cannot be considered a highly oxidized matrix, due to its relatively low PUFA content, meat and meat-products undergo oxidative changes during storage, processing, digestion, and metabolization, which make them a potential source of oxidizing agents. These changes take place from the moment of the animal’s slaughter, where the conversion of the muscle into meat already begins to form oxidation compounds. The main substrates susceptible to oxidation are lipids, especially those long-chain PUFAs, which provide the food with variations in its texture, color, flavor, and odor. All these oxidation reactions follow a common process: an initiation stage, in which free radicals are generated; a propagation phase in which the number of oxidized and oxidizing compounds multiplies; and finally, a third stage that is known as a termination, in which the radicals react with each other or with other non-radical compounds (antioxidants) to give rise to non- or less-oxidizing products. The mechanisms involved in lipid oxidation have been elegantly reported elsewhere [51].

**Figure 1:**
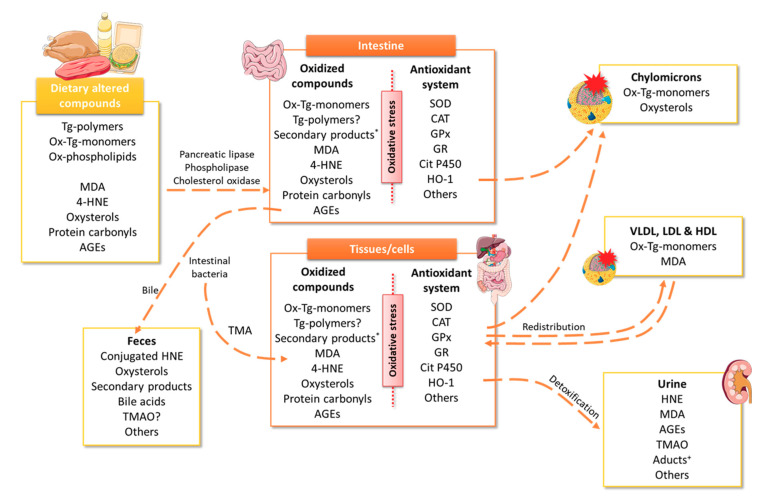
Possible metabolic fate of dietary altered compounds. The scheme illustrates the different body pathways for eliminating those altered products observed. *secondary products included short- or medium-chain alkyl group, carbonyl, hydroxyl, aldehyde, ester, epoxy, carboxyl, etc. ^+^Aducts include HNE adducts and HNE conjugates. 4-HNE, 4-hydroxy-nonenal; AGEs, advanced glycation end-products; CAT, catalase; GPx, glutathione peroxidase; GR, glutathione reductase; HDL, high-density lipoproteins; HNE, hydroxynonenal; HO, heme oxygenase; LDL, low-density lipoproteins; MDA, malondialdehyde; Ox, oxidized; SOD, superoxide dismutase; Tg, triglycerides; TMAO, trimethylamine N-Oxide; VLDL, very low density lipoproteins.

On the other hand, a high intake of meat and meat-products can promote reactive oxygen species (ROS) formation at the gastrointestinal tract [14]. Many of these products are generated from lipid hydroperoxide decomposition such as reactive aldehydes, ketones and epoxides, which are cytotoxic [52]. Once formed in the stomach, they are easily absorbed by the intestine to subsequently interact with proteins and lipids to form advanced lipid oxidation end-products [52]. It has been shown that animals and humans, after ingesting peroxidized foods, absorb and excrete large amounts of MDA, 4-HNE, and other carbonyls (Figure 1). The pathological effects of reactive aldehydes are related to their ability to modify reactive proteins or DNA by cross-linking, protein oligomerization, immune responses, and to bind to the receptor for advanced glycation end-products (AGEs), activating the NADPH oxidase and generating ROS [52].

**Figure 2:**
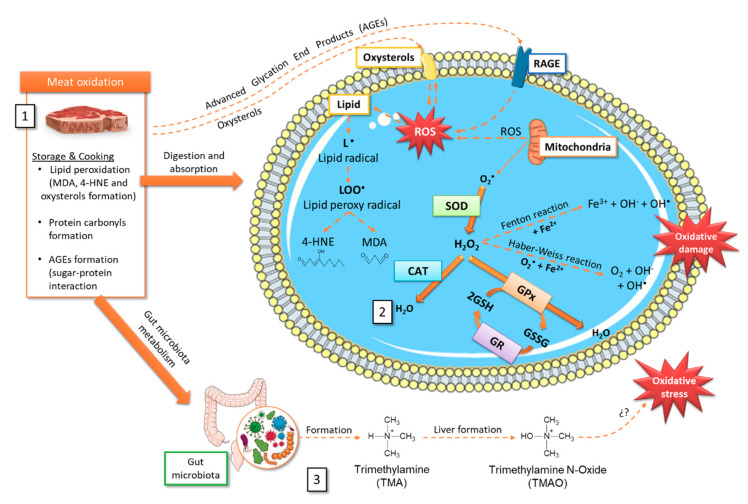
Tentative scheme relating storage and cooking oxidative processes in meat and the production/elimination of radical oxygen species (ROS). It is detailed (1) Oxidized compounds that can be generated in meat during storage and cooking. (2) Some intracellular mechanisms involved in ROS formation, oxidative damage, and possible elimination through the antioxidant machinery. (3) TMAO formation after choline and L-carnitine metabolism by the gut microbiota and subsequent oxidation in the liver. 4-HNE, 4-hydroxy-nonenal; AGEs, advanced glycation end-products; CAT, catalase; GPx, glutathione peroxidase; GR, glutathione reductase; GSH, reduced glutathione; GSSG, oxidized glutathione; H_2_O_2_, hydrogen peroxide; LOO•, lipid peroxyl radical; MDA, malondialdehyde; RAGE, receptor for AGEs- advanced glycation end-products; ROS, radical oxygen species; SOD, superoxide dismutase; TMA, trimethylamine; TMAO, trimethylamine N-Oxide.

In this section, the main oxidation compounds generated during the storage, cooking or digestion process will be briefly summarized; among which are MDA, 4-HNE, oxysterols, protein carbonyls, advanced glycation end-products (AGEs), and trimethylamine N-oxide (TMAO), that, once consumed, can induce oxidative stress (Figure 1 and Figure 2).

### 4.1. Heme Iron

As previously commented, one of the first links in the oxidative chain processes is iron. This mineral is present in the diet as heme iron, derived from animal origin foods, and non-heme iron, in plant and animal origin sources [53]. Heme iron in meat is highly variable depending on the species, there being a greater amount in beef than in pork or chicken [8]. Heme iron constitutes myoglobin and hemoglobin, not being especially dangerous in oxidation terms. However, during the muscle into meat transformation, storage or digestion/metabolization, changes associated with oxygen depletion and decreased pH predispose heme iron to act as a pro-oxidant [42]. 

Iron presents the ability to alternate between oxidation states when accepting and donating electrons easily, changing from ferric (Fe^3+^) to ferrous (Fe^2+^) and vice versa (Figure 2). Given its oxidizing potential, we found that this mineral is normally bound to proteins for its storage and transport (forming complex with ferritin, transferrin or other proteins as hemoglobin, myoglobin, cytochrome c, cytochrome P450, nitric oxide synthases or guanylate cyclase) [54] that partially or almost completely blocked its oxidation. However, when heme iron is free it can cause oxidative damage since it rapidly loses the heme porphyrin ring and contributes in the ferrous state to ROS generation through Fenton reaction. A high heme iron intake can induce lipid peroxidation (LPO) in two main ways: in the initiation stage, catalyzing the ROS formation; or in the propagation phase, catalyzing the peroxide decomposition to promote LPO. Heme iron catalyzes the superoxide and hydrogen peroxide conversion to hydroxyl radicals (OH•) by the Fenton reaction (Figure 2) [44]. Due to its lipophilic nature, heme toxicity is further exacerbated by its ability to insert itself into lipid membranes and to catalyze cell membrane oxidation, with the subsequent cytotoxic lipid peroxide formation which can lead to cell death [55]. It can also act as a nitrosating agent after being metabolized by intestinal bacteria and generate N-nitroso compounds, capable of causing DNA damage or DNA adducts formation [56]. In addition, heme iron can also participate in lipoprotein oxidation and lipid peroxidation end-products formation, such as MDA, 4-HNE, oxysterols, and aldehyde during meat storage, processing, and digestion [56].

Iron absorption and metabolism are tightly regulated in vivo in an attempt to prevent reactive iron species from participating in uncontrolled oxidation reactions [54,57]. Iron is absorbed by the enterocyte apical membrane in the form of heme iron or as Fe^2+^ and Fe^3+^ being rapidly metabolized for storage as ferritin or exported through ferroportin. An intracellular heme iron overload can affect the redox state, where the enzyme heme oxygenase (HO), especially the inducible form HO-1, plays an essential role in breaking it down to produce biliverdin, carbon monoxide, and Fe^2+^ [54,55]. Biliverdin is rapidly reduced by biliverdin reductase to produce bilirubin, which can effectively remove peroxyl radicals, thereby inhibiting LPO, attenuating heme-induced oxidative stress, cell activation, and death [55]. Likewise, the released carbon monoxide activates nuclear factor E2-related factor 2 (Nrf2) signaling, promoting the endogenous antioxidant availability and increasing HO-1 levels [58]. On the other hand, ROS stimulate ferritin synthesis and iron sequestration, being essential for proper iron homeostasis [55]. Therefore, heme iron can contribute to oxidative processes both in meat and in the organism once consumed. However, within healthy nutritional patterns, the endogenous antioxidant system is capable of alleviating and also blocking the oxidative effect induced. Again, several different situations made this topic complex and difficult to study and clearly demand to avoid doing any generalization as iron content in meat is rather variables as we have discussed in Section 4. In addition, the consumption of “red” meat is expected to induce more oxidation effects after a large interprandial period (e.g., 15 h) that after a shorter one (4–5 h) as the activity and expression of gastrointestinal antioxidant enzymes is highly modified by the fasting conditions [59].

**Figure 3:**
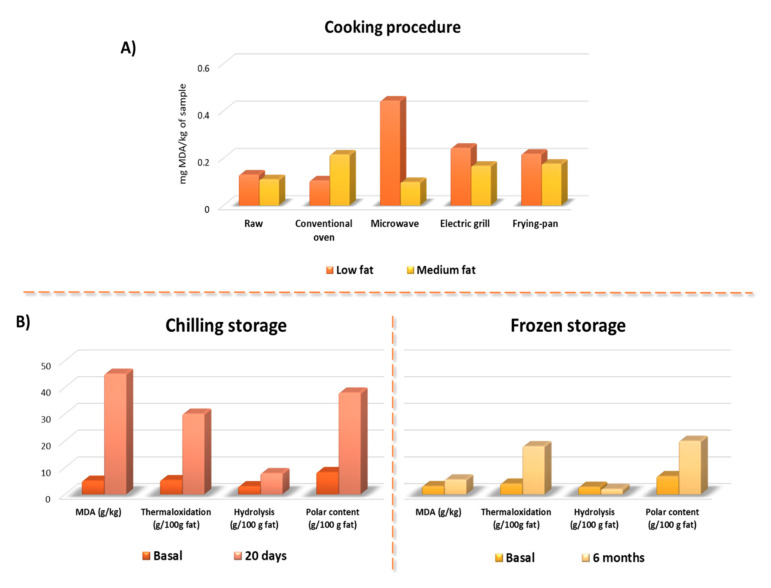
Oxidation compounds formation according to the cooking and storage method. (**A**) Results of the MDA formation in beef steaks with low and medium fat content subjected to different cooking methods. (**B**) Oxidation compounds formation in preserved meat in chilling or frozen storage. Modified from Serrano et al. [62] and Librelotto et al. [64]. With Meat Science permission solicited and still pending.

We also have to keep in mind that most studies have been performed in raw meat or in in vitro model conditions, with no final conclusions on the importance of cooking and the type of cooking on meat oxidation. More, researches on the interaction of cooking–digestion oxidations are scarce [60]. When heated, Fe^2+^ is released by the heme-porphyrin moiety destruction, oxymyoglobin releases O_2_, leading to H_2_O_2_ production, and antioxidant enzymes (such as glutathione peroxidase) are inactivated [60]. These reactions favor the stimulation of the Fenton reaction, which leads to greater LOP formation. On the other hand, several cooking methods can be applied to cook meat in order to increase its acceptability and digestion, which in turn modify original composition and give rise to oxidation. The effect of cooking on lipid oxidation has been related to the warmed-over flavor in cooked meats. The explanations offered take into account factors associated with the thermal treatment itself (cooking methods, final cooking temperature or cooking rate) and with the product composition (amount and type of lipid, antioxidant, etc.) [61]. In a previous study, meat lipid oxidation was evaluated by monitoring secondary oxidation compounds (thiobarbituric acid reactive substances—TBARS) [62]. When specific cooking procedure (conventional oven, microwave oven, electric grill, and pan-frying) were applied to same restructured beef meat formulated to present low or medium fat content the TBARS changed. However, results suggest that oxidation was low in all restructured steaks (Figure 3). Irrespective of product formulation, the cooking process did not always cause an increase in TBARS values; however, the most important changes in lipid oxidation induced by cooking were observed in low fat samples, mainly those cooked in a microwave oven (Figure 3A). Microwaving has been reported to induce increased thermaloxidation in oils [63]; however, this effect was not observed in medium fat steaks. Similar effects were observed in pan-frying and grilling [64]. Thus, in some way it seems that the fat presence exerts some kind of protection against oxidation. It could be speculated that fat avoid the releasing of Fe^2+^ in low fat samples but this hypothesis was not evaluated. 

Recently we have published the importance of frying on Mediterranean cousin, technique that contrarily to that it is though permits to improve food fatty acid composition and stability depending on the oil type used [65]. In fact, seed oils are more susceptible of thermaloxidation that monounsaturated oils, determining that food cooked with seed oils contain higher amounts of these undesirable compounds [66], thus contributing, as in the specific case of Milanese, a typical Italian heritage-Mediterranean food, to some potential negative effects of meat consumption. Again, we think that several aspects are still pending to be more deeply studied.

### 4.2. Malondialdehyde and Polar Triglycerides

MDA is an organic compound resulting from the LPO of arachidonic acid and other long-chain PUFAs through enzymatic and non-enzymatic processes [67]. It is important to note that during meat processing and storage, the MDA formation takes place through non-enzymatic processes, derived from lipid peroxidation. However, in a living organism, the MDA formation can be produced both by a non-enzymatic mechanism and by enzymatic processes during the biosynthesis of thromboxane A 2 (TXA 2) and 12-l-hydroxy-5,8,10-heptadecatrienoic acid (HHT) [68]. MDA is one of the most abundant and well-known aldehydes generated during secondary lipid oxidation and is also the most widely used as an oxidation marker in meat and meat-products. Once consumed, MDA can be enzymatically metabolized, or it can interact with proteins, nucleic acids and lipids, being able to alter a great variety of biological molecules [69]. Lipoproteins are also more susceptible to oxidation after red meat consumption, which is a major problem for CVD. It can establish covalent modifications within lipoproteins, especially with LDL, as observed in studies in humans where higher plasma MDA levels and oxidized-LDL after meat chops consumption were found in comparison to its turkey meat counterpart.

Current evidence suggests the presence of high plasma MDA levels after red meats or processed products consumption by healthy individuals [70]. This could be due, partially, to the fact that the stomach acts as a bioreactor, generating a large amount of ROS and promoting the LPO. Studies of in vitro digestions have demonstrated that the meat fatty profile is a determinant for the MDA formation in its passage through the gastrointestinal tract [49]. Although no clear explanation is available, high iron levels seem to contribute to oxidation at the stomach [71].

Polar material determination has proven to be one of the most specific methods to analyze fat and oil alterations [72]. The polar material content consists of glycerides and free fatty acids with higher polarity than those the original triglycerides, as polymers of triglycerides, dimers of triglycerides; oxidized triglycerides; diglycerides; monoglycerides; and free fatty acids. Based on our group large experience on evaluating fat oxidation by column chromatography followed by high-pressure size exclusion chromatography [72], the meat fat alteration was evaluated in restructured pork models submitted to chilling and frozen storage [10]. MDA was also determined by meaning of the TBARS analysis. TBARS, thermal oxidized compounds (polymers of triglycerides, dimers of triglycerides; oxidized triglycerides) as well as hydrolytic compounds (diglycerides; monoglycerides; and free fatty acids) increased significantly during storage. Several compounds affected oxidation in the tested meat systems (a) relatively high fat percentages, which generally promote oxidation susceptibility; (b) prepared with added NaCl, and salting that is known to increase the prooxidant activity of iron in myofibrillar foods; (c) non addition of some common additives with antioxidant activity (e.g., nitrites, ascorbic acids, etc.); (d) major reduction in particle size (and hence loss of structural integrity) which increased the exposure of labile compounds to oxygen; (e) heating during meat system preparation (70 °C) [10]. A detailed observation of results suggests that fat alteration after 6-month chilling was about the half that that observed after 20 days at chilling conditions (Figure 3B). 

### 4.3. 4-Hydroxy-Nonenal

4-HNE is a secondary oxidation product from LPO and potentially has genotoxic properties [49]. 4-HNE has been extensively studied, not only for its function as a signaling molecule that stimulates gene expression, but also for its cytotoxic role that inhibits gene expression and promotes the development and progression of different pathologies. 4-HNE can be formed endogenously through enzymatic and non-enzymatic processes, although considerable levels have also been found in foods. Similar to MDA, the 4-HNE formation by enzymatic processes is due to the metabolism of ω-6 PUFA by 15-lipoxygenase. On the other hand, in the non-enzymatic formation of this oxidation product, five possible routes have been described, for which it is recommended to review Ayala et al. [68]. In meat-products, after interacting with its histidine residues, 4-HNE promotes myoglobin redox instability, accelerating meat discoloration and inducing LPO [73]. This covalent modification induces iron liberation from the pigment, facilitating further meat worsening [74]. 4-HNE is also known as a second oxidative stress messenger and has a longer half-life than ROS (Figure 2). Its possible involvement in numerous pathological processes such as metabolic diseases, neurodegenerative diseases, and cancers has been observed, probably due to its chemical reactivity and ability to form covalent adducts with biological molecules [68]. 

As positive association between heme iron levels and 4-HNE formation has been revealed in both animal diets and meat-products, it seems crucial to control the 4-HNE presence in the diet, mainly as meat-products or foods rich in ω-6 PUFA or selenium. Metabolism and the subsequent 4-HNE elimination is predominantly done through the glutathione system by reduced glutathione (GSH) conjugation by glutathione-S-transferase to originate the conjugate GSH-4-HNE although there are also other alternative pathways involving aldehyde dehydrogenase and alcohol dehydrogenase enzymes. Once inactivated, 4-HNE is eliminated as conjugated metabolites through urine or bile (Figure 1) [75].

### 4.4. Oxysterols

Cholesterol is a noteworthy component of meat and meat-products. Given its lipid nature, it is susceptible to oxidation during the production and storage process, giving rise the dreaded oxysterols or cholesterol oxidation products, whose levels in fresh products are very low [76]. These oxidative modifications involve the addition of one or more oxygen-containing functional groups on the sterol B ring and/or on the side chain. Of the different oxysterols that can originate in food, the most common are 7-oxygenated sterols and 5α,6α-oxygenated sterols [77]. The average oxysterols amount present in meat and meat-products varies from 0.1 to 18.7 µg/g [76]. However, these concentrations may modify depending on the heat treatment, the type and duration of storage and the meat matrix composition [77,78]. The oxysterols formation is a temperature-dependent process, as dramatic increase in foods cooked to high temperatures was observed [79,80]. On the other hand, as with the vast majority of lipids, light and the oxygen presence are key factors in oxidative processes, both conditioned by exposure time [78]. Furthermore, all these processes are influenced by the lipid composition of the meat-matrix, with a greater formation of oxysterols being observed in PUFA-rich meat-products [76,81].

The presence of oxysterols in the body can be of endogenous origin (mainly derived from non-enzymatic processes) or exogenous, from the diet. Although oxysterol absorption rate is lower than that of cholesterol, there is a direct correlation between the amount of oxidized lipids ingested and plasma oxysterols levels [82,83]. These compounds are absorbed in the upper digestive tract and are transported by chylomicrons and other lipoproteins, being able to deposit them in peripheral tissues and increase oxidative stress. They are generally eliminated through bile, after metabolization by the liver (Figure 1) [77,84]. Various studies have suggested that oxysterols are potentially involved in the onset and progression of chronic diseases such as atherosclerosis or T2DM since they have proinflammatory, cytotoxic, and mutagenic properties [76,85].

### 4.5. Protein Carbonyls

In addition to lipid substrates oxidation, it is currently known that proteins in meat-products can be a source and target of ROS [86]. These alterations had gone unnoticed until recently when oxidized proteins were suggested to have a negative impact on meat quality [87,88]. Numerous studies reported that protein oxidation takes place during the post-mortem stage, meat handling, processing, and storage [86]. However, current evidence largely ignores how oxidative processes take place from proteins, peptides, and amino acids. What does seem evident is the interaction between lipids and oxidized proteins lead to a marked meat deterioration [89,90].

Protein oxidation results from a chain reaction similar to that occurring in lipids, in which superoxide, hydroperoxyl and hydroxyl radicals, hydrogen peroxide, hydroperoxides, peroxyl radicals, and heme iron seem to be the triggering factors. The level and nature of the oxidation products formed largely depends on the amino acids involved and how the oxidation process begins [86,91]. Among the possible modifications that may be suffered, the formation of protein carbonyls (aldehydes and ketones) is the most prominent and has become the most common measurement to evaluate protein oxidation in meat and biological systems because it is easily measurable through the 2,4-dinitrophenylhydrazine (DNPH) technique [92,93]. Carbonylation is an irreversible and non-enzymatic modification of proteins, which mainly takes place from the direct oxidation of the lysine, threonine, arginine, and proline side chains. Other possible oxidation routes have also been described, such as enzymatic non-glycation in the presence of reducing sugars, oxidative cleavage of the peptide skeleton through the α-amidation pathway or by oxidation of glutamyl side chains and covalent binding to compounds of non-protein carbonyl such as MDA or 4-HNE [86,94] whose origin and relevance has been already discussed in previous subsections.

Furthermore, studies have shown that oxidized components consumption in the diet increases oxidation markers in both blood and tissues, promoting oxidative stress. Despite the fact that most of the available literature has focused on the effect of oxidized lipids consumption, some studies indicated that oxidized protein intake increases oxidative stress both in blood and in the digestive tract of mice [95,96]. In line with these data, other authors already establish a relationship between its consumption and the development of chronic diseases such as T2DM [94,97,98].

### 4.6. Advanced Glycation End-Products

AGE products are compounds generated from a non-enzymatic reaction between reducing sugar and the amino groups of proteins, lipids, or nucleic acids, traditionally known as the Maillard reaction [99]. AGEs are highly oxidizing compounds that are also known as glycotoxins and can be formed endogenously or ingested through the diet [100]. These same authors have developed a database where they summarized the AGEs amount in food, with a special mention for meat and meat-products and suggest that due to its high consumption meat-products could highly contribute more to AGEs daily intake. Furthermore, the AGEs formation is notably increased by high temperatures cooking, one of the most common practices in meat preparation [100]. 

High consumption of AGEs has been related to CVD, kidney disease, and especially with T2DM [100,101,102]. AGEs present relatively high bioavailability as 10% of the consumed AGEs are absorbed in the intestine and contribute to the AGEs systemic levels and the formation of ROS and increase oxidative stress (Figure 1 and Figure 2) [102]. Their pathological effects have been related to their ability to promote oxidative stress and proinflammatory cytokines production. In addition, AGEs can induce oxidative stress by interacting with the insulin receptor, whose union has been shown to increase ROS production through NADPH oxidase [103]. Moreover, it was shown that AGEs upregulate the receptor for AGEs (RAGE) expression in various cell types, entering a vicious circle and inducing the transcriptional factor nuclear factor-κB (NF-κB) sustained activation [101].

### 4.7. Trimethylamine N-Oxide

Currently, research on meat consumption focuses on the study of TMAO. This is an organic compound generated by intestinal bacteria from choline and L-carnitine rich sources. Fundamentally, choline is absorbed in the small intestine, but when there is excessive supply—as occurs after there has been a high red meat intake—, it reaches the large intestine and is metabolized by the microbiota to produce trimethylamine. Besides, L-carnitine undergoes bacterial processing similar to choline. In both cases, the trimethylamine formed oxidizes rapidly in the liver and gives rise to TMAO (Figure 2) [104]. Different metaanalyses have established a strong relationship between elevated circulating TMAO levels and CVD risk [105]. In fact, a high red meat intake increases the systemic TMAO levels in healthy humans, mainly due to the greater availability of dietary precursors, the TMAO bigger formation by the microbiota and the lower renal excretion [106]. Several studies have found that plasma TMAO levels can affect lipid homeostasis by modulating cholesterol metabolism [107]. Likewise, recent research has shown that TMAO can play an important role in endothelial dysfunction and in atherosclerosis development since it promotes oxidative stress by increasing ROS and MDA levels and reduces the superoxide dismutase (SOD) levels. Similarly, it also participates in vascular inflammation by inhibiting the expression and activity of eNOS, reducing nitric oxide production, increasing NF-κB signaling, inducing inflammasome activation and the release of proinflammatory cytokines [108,109,110,111].

## 5. Practical Implications and Future Works

Several scientific societies have called for the need to consume a plural diet in which both animal and vegetable foods should be present to diminish potential deleterious effects derived from subclinical deficiencies and bad storage and cooking practices. Apart from its nutritional composition, one of the major advantages of meat and meat-products is being a good matrix to include functional ingredients that, in addition to improving meat composition and health properties, they would contribute changing the currently negative image attributed to this food-group [2,19,20,21]. Research and development of functional meat should take advantages of these previous premises in order to employ the most convenient meat, under a health point of view, to incorporate health ingredients. However, the need for strict controls of the ingredient quality used in functional meat design and preparation has to be pointed out in order to avoid the presence of undesirable contaminants or inadequate composition. As meat is normally storage at a low temperature before consumption and submitted later to different cooking treatments, active research is demanded to increase knowledge of reliable deleterious effects on oxidation.

As for other foods, the potential negative interactions with other dietary compounds (e.g., food, nutrients) have to be tested for regular and functional meats. The concern is focused on drugs that are metabolized by cytochrome P450 isoenzymes, as we have commented on, how several ingredients (e.g., polyphenols) affect this metabolizing enzyme highly modifying or even reversing the antioxidant properties of a given functional meat to a pro-oxidant one. 

Although growing evidence exists, most obtained data come from in vitro studies being urgent to perform in vivo studies in animals and human. Individual response to therapeutic diets is striking which appears to have a genetic component [16]; thus, genetic factors (presence of risk allele) affecting dietary response to antioxidant ingredients of functional meat should be investigated in order to get more targeted and potentially efficient functional meats for the prevention or amelioration of oxidation and their deleterious consequences [111]. Therefore, the presence of alleles negatively contributing to a lower antioxidant activity of GR, GPx, NO syntase, parooxonase (PON-1) or to a higher prooxidant activity induced by cyclooxygenase, lipoxygenase and different isoforms of cytochrome P450 in some individuals should be investigated by meaning of GWAS and EPIWAS trying to get a GRS (Gene-risk score) that advances the understanding of the reality of nutrigenetics on functional foods addressed to improve antioxidant status [112]. As colonic microbiota highly contributed to systemic oxidation [113], the effects of functional foods on microbiota diversity, abundance, genomics characteristics (metagenomics) and antioxidant/pro-oxidant activities should be investigated, with the use of the new Omics modalities/sciences/strategies: transcriptome, proteome, metabolome, metagenome being necessary for a more precise design of functional meat and its application to precision diets. Finally, we want to highlight the importance of personalized nutrition, since, as suggested by Van Hecke et al., a high consumption of red and processed meat may present a higher risk for some population subgroups, for example, individuals infected with *Helicobacter pylori* those with inflammatory bowel disease, which are common gastrointestinal diseases associated with oxidative stress [8]. Likewise, the intestinal microbiota can also play a key role in the possible involvement of degenerative diseases associated with oxidative stress.

Animal studies have reported that eating processed meat promotes proteolytic bacteria growth associated with non-alcoholic fatty liver disease and obesity [114,115,116]. It is also known that a relatively high proportion of ingested heme iron reaches the colon, which could stimulate oxidation reactions in the colon, especially when large amounts of red meat are ingested [116]. In fact, the increase of oxidized lipids in colonic tissues is one of the proposed mechanisms that relate red meat consumption to colorectal cancer development [117,118]. All these processes are interconnected so that an excessive arrival of oxidized products derived from meat could induce intestinal dysbiosis, alter its metabolic capacity, and facilitate deleterious effects on colonic mucosae [119,120]. Some authors suggest the hypothesis that this relationship could be due to heme iron-induced dysbiosis since a greater presence of bacteria considered pathogenic (e.g., *Streptococcus bovis*, *Bacteroides*, *Enterococcus faecalis*, *Escherichia coli*, and *Clostridia*) in the presence of this mineral has been observed [121]. Well-controlled studies have to be performed both in animal models and humans to find out if all these processes are interconnected.

## 6. Conclusions

Meat and meat products are amply consumed all over the word, significantly contributing to people’s nutrition.

Low to very low meat consumption makes it highly difficult to cover dietary recommendations, particularly of vitamins B_6_ and B_12_. 

Several cuts (pieces) of terrestrial and marine mammals, differing in species, gender, age, livestock practices, and feeding, are included in the concept of meat.

There are large dissimilarities (e.g., fat infiltration, fatty acids, and fat-soluble vitamins content and composition) between meat from monogastric and polygastric animals. 

Their consumption highly exceeds the daily amount considered adequate (mainly due to the high SFA and heme iron contents) and because of this, high intake induces the low consumption of other food-groups conditioning health status.

At present, no consensus on the concept and differences between red and white meat is available. 

Iron and PUFA meat contents highly contribute to meat oxidation during storage and cooking.

Meat and meat-products oxidation give rise to increased MDA and polar material, 4-HNE, oxysterols, protein carbonyls, AGEs, and TMAO levels contributing to the deleterious effects ascribed to high meat consumption. 

Meat and meat-products digestion and metabolization contribute to body oxidation status. 

Long-term intervention-controlled studies testing different types and amounts of meat and meat-products are demanded to accurately ascertain the relationship between meat, oxidation, and degenerative diseases.

## Figures and Tables

**Table 1:** Meat consumption worldwide and by continent in 2017.

Type of Meat	Meat Consumption (kg/per capita/year) ^*^					
	World	Africa	America	Asia	Europe	Oceania
Bovine	9	5.63	27.83	4.68	14	31.22
Mutton and goat	1.86	2.49	0.62	1.93	1.75	10.79
Pork	15.7	1.48	18.65	15.18	35.75	24.24
Poultry	15.18	6.21	41.94	9.71	24.59	43.96
Others	0.84	1.43	0.65	0.55	1.84	2.1
Total	42.58	17.24	89.69	32.05	77.93	112.31

^*^ The per head supply of each such food item available for human consumption is then obtained by dividing the respective quantity by the related data on the population actually partaking of it (FAOSTAT). Most recent data available in this database [28].

**Table 2:** Nutritional composition of main types of red meat (beef and pork) in USA, UK, and Spain.

	Beef			Pork		
	USA	UK	Spain	USA	UK	Spain
Energy (kcal)	126	129	131	144	124	155
Protein (g)	21.0	22.5	20.7	21.2	21.8	20.0
Fat (g)	4.0	4.3	5.4	5.9	4.0	8.3
SFA (g)	1.4	1.7	2.2	2.0	1.4	3.2
MUFA (g)	1.6	1.9	2.5	2.7	1.5	3.6
PUFA (g)	0.2	0.2	0.2	0.6	0.7	0.6
Niacin (mg)	6.2	9.7	8.1	4.8	6.9	8.7
Tiamin (mg)	0.1	0.1	0.1	1.0	1.0	0.9
Vitamin B_12_ (μg)	1.5	2.0	2.0	0.7	1.0	3.0
Iron (mg)	1.8	2.7	2.7	0.9	0.7	1.5
Zinc (mg)	3.9	4.1	3.8	2.0	2.1	2.5
Selenium (mg)	26.0	7.0	3.0	32.4	13.0	14.0
Sodium (mg)	54.0	63.0	61.0	54.0	63.0	76.0
Potasium (mg)	323.0	350.0	350.0	384.0	380.0	370.0

Data related to 100 g edible meat. SFA, MUFA, PUFA, saturated, monounsaturated, and polyunsaturated fatty acids. Modified from Delgado-Pando [40].

**Table 3:** The label of red and white meat according to iron content.

	Food and Nutrient Database for Dietary Studies			Base de Datos Española de Composición de Alimentos				
	Iron (mg)	Total Fat (g)	SFA (g)	PUFA (g)	Iron (mg)	Total Fat (g)	SFA (g)	PUFA (g)
Iberian ham	14.29	25.0	7.14	1.31	4.3	19.2	7.81	1.18
Pâté	9.19	13.1	4.0	2.46	5.5	29.5	10.48	3.59
Ground beef	1.97	19.07	7.29	0.51	1.9	21	8.51	1.25
Ground lamb	1.78	19.49	8.05	1.39	1.12 *	14.55	5.34	0.74
Ground Pork	1.28	20.6	7.66	1.85	1.3	23	7.43	3.51
Pork sausage	1.2	27.25	8.83	5.12	1.44	28.1	10.55	2.60
Cooked ham	1.0	17.46	6.42	1.67	1.2	5.1	1.9	0.6
Turkey leg	0.83	3.38	1.0	0.91	1.5	8.36	2.6	2.3
Chicken nuggets	0.83	20.36	3.57	6.51	1.39	15.12	2.88	2.3
Chicken breast	0.51	7.67	1.96	0.95	1.5	1.2	0.33	0.28

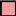
 Red meat and meat-products; 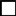 White meat and meat-products. * mostly suckling lamb; PUFA, polyunsaturated fatty acids; SFA, saturated fatty acids; Source: Food and Nutrient Database for Dietary Studies (FNDDS) and Base de Datos Española de Composición de Alimentos (BEDCA). Compositions are expressed either per 100 g edible portion of different meat.

## Abbreviations

4-HHE: 4-hydroxy-hexenal; 4-HNE, 4-hydroxy-nonenal; AGEs, advanced glycation end-products; CAT, catalase; CVD, cardiovascular diseases; DNPH, 2,4-dinitrophenylhydrazine; GPx, glutathione peroxidase; GR, glutathione reductase; GRS, gene-risk score; GSH, reduced glutathione; GSSG, oxidized glutathione; HDL, high density lipoproteins; HO, heme oxygenase; LDL, low-density lipoproteins; LPO, lipid peroxidation; MDA, malondialdehyde; MUFA, monounsaturated fatty acids; NF-κB, factor nuclear factor-κB; Nrf2, nuclear factor E2-related factor 2; PUFA, polyunsaturated fatty acids; PON-1, paraoxonase-1; RAGE, receptor for AGEs- advanced glycation end-products; ROS, reactive oxygen species; SFA, saturated fatty acids; SOD, superoxide dismutase; T2DM, type 2 diabetes mellitus; TBARS, thiobarbituric acid reactive substances; TMAO, trimethylamine N-Oxide; VLDL, very low density lipoproteins.
